# The League of Mercy

**Published:** 1917-12-29

**Authors:** 


### THE LEAGUE OF MERCY.

### Eighteenth Annual Meeting of Presidents.

### Conferment of the New Bar of the Order.

The eighteenth annual meeting of Presidents of the League of Mercy was held on Friday, December 21, at St. James's Palace, the Right Hon. Viscount Farquhar, G.C.V.O., presiding. Supporting him on the platform were H.H. Princess Marie Louise, who made the presentations, and the Speaker of the House of Commons.

His Majesty's Letter.

The **Chairman** announced that H.M. the King had sent to him, through Lord Stamfordham, the following letter: "My dear Farquhar,—I am commanded by the King and Queen to express to the meeting of the Presidents their Majesties' gratification at the result of this year's work of the League of Mercy. A sum of £15,000 has again been handed over to King Edward's Hospital Fund for London, and more than £3,000 distributed to hospitals outside the Metropolitan area. The total amount of £285,000 has been contributed by the efforts of the League to the voluntary hospitals since its foundation. In their Majesties' opinion, this sustained activity, despite many difficulties, reflects the highest praise on all its officers and workers. The King has approved the institution of a Bar, to be awarded to those who shall, for a long period of years, have continued good services to the League subsequently to having received the Order of Mercy. His Majesty has been graciously pleased to award the first Bar to Dora Countess of Chesterfield, Lady President of South Kensington, whose district stands at the head of the list with the largest aggregate collection to its credit from 1899 to the present time.—Yours very truly, Stamfordham."

**Lord Farquhar** congratulated the Countess of Chesterfield cordially on the honour. He regretted the absence of the Grand President, the Earl of Athlone, on account of military duty abroad, and the absence of H.R.H. Princess Alice, who had always been most constant in the work of the League. The meeting was, however, to be congratulated on the presence of H.H. Princess Marie Louise, who would kindly hand the awards of the Order to those who had earned them. The gathering was also fortunate in being able to welcome Mr. Speaker, one of the Governors of King Edward VII.'s Hospital Fund, who would give an address.* This was the third year that a state of war had existed, and seeing what an amount of energy and work had been put into the collections, it was gratifying and remarkable that last year's total had been improved upon. £19,971 was received last year, out of which £15,000 was given to King Edward VII.'s Fund (£1,000 more than the year before) and £3,193 was given to county hospitals. People seemed to have recognised that the League was itself a war charity, by reason of the help it gave to the voluntary hospitals, and its signal services to the many thousands of wounded and incapacitated soldiers. An important feature this year was the bazaar which was held in October at the Ritz Hotel, which resulted in an addition of £1,700 to the funds after paying all expenses, and he thanked all concerned for their contribution towards that gratifying result. He specially mentioned in that regard the names of Miss Olive Andrews and Mr. Walter Andrews, who were responsible for much of its organisation. The restaurant scheme was again taken up, and proved a great success. The League of Mercy Film had been pursuing its wonderful career round the country, and the house-to-house collections had been a success in many districts. Moreover, meetings in various parts of the country to further the interests of the League had been held, and had been well attended. He hoped such meetings would be held in every district throughout the coming year. A very good instance of the result of meetings was afforded by the case of Oxfordshire, where a collection of £80 had this year been increased to £144. None of the charitable agencies got in their collections at such a small expense as did the League. He congratulated all the Presidents on their work, and specially named the Secretary, Mr. Franklyn, and the Assistant Secretary, Miss Milnes.

* This is regretfully held over owing to pressure on our space.—Ed. The Hospital.

**Sir Frederick Green,** the Hon. Treasurer, gave a short *résumé* of the work, naming the localities where markedly improved results had been attained. The actual total for the year was £21,022, an increase on last year of £1,050. Lady Chesterfield's district—South Kensington—collected £1,375. It was a source of great regret that the Countess was likely to relinquish the work, but if she was not able to re-consider her decision, it was earnestly hoped that the League might at times be favoured with her advice. West Marylebone had collected £1,200, the £1,700 realised by the Bazaar at the Ritz Hotel having been allotted to the separate districts of the stallholders. South Paddington—Lady Dimsdale's district, £934. He mentioned that a Maori sergeant collected and sent 200 francs from Maoris in the front-line trenches, 25s. was received from a hospital in Cairo, and a supporter in British East Africa sent £10 which he had collected.

**Sir William Collins** followed, and the formal votes appointing the honorary officers and the members of the Executive Committee were spoken to by Sir Owen Philipps, Sir Charles Wakefield, and Mr. Montagu Sharpe, J.P.

**THE SPEAKER'S ADDRESS in full will be published in "The Hospital" of January 5, 1918.**

